# Supplementary material for: Cryo-EM structure of a conjugative type IV secretion system suggests a molecular switch regulating pilus biogenesis
Source: EMBO J. 2024 Jun 17;43(15):10. doi: 10.1038/s44318-024-00135-z (PMC11294453; doi:10.1038/s44318-024-00135-z)
Supplement: Supplementary file 1 — Table EV1 [file 44318_2024_135_MOESM1_ESM.docx]

**Table EV1.**

**A**. Cryo-EM data collection and processing statistics and Expanded View information. The EV information part of this table provides information on how each map was used, the part(s) that were built in each map when relevant, the method that was used to build the model from each map when relevant, and the PDB entry code of the corresponding model.

| Complex/Subcomplex |  | **OMCC** | | | | |  | **STALK-ARCHES-IMC** | | | |
| --- | --- | --- | --- | --- | --- | --- | --- | --- | --- | --- | --- |
|  |  |  |  |  |  |  |  |  |  |  |  |
| Name of the maps generated in this study |  | O-Layer C14 at 2.5 Å | I-Layer C16 at 2.7 Å | Conformation-A at 3.2 Å | Conformation-B at 2.9 Å | Conformation-C at 3.1 Å |  | Stalk C5 at 3.0 Å | Arches at 6.2 Å | Extended IMC protomer at 3.8 Å | STALK-ARCHES-IMC at 4.3 Å |
| EMDB entry |  | EMD-19478 | EMD-19479 | EMD-19480 | EMD-19481 | EMD-19482 |  | EMD-19483 | EMD-19484 | EMD-19485 | EMD-19488 |
|  |  |  |  |  |  |  |  |  |  |  |  |
| **Data collection and processing** |  |  |  |  |  |  |  |  |  |  |  |
| Magnification |  | 130,000 | | | | | | | | | |
| Voltage (kV) |  | 300 | | | | | | | | | |
| Electron exposure (e^–^/Å^2^) |  | 54 | | | | | | | | | |
| Defocus range (μm) |  | -1.5 to -3.5 | | | | | | | | | |
| Pixel size (Å) |  | 1.067 | 1.067 | 1.067 | 1.067 | 2.134 |  | 1.067 | 1.067 | 1.067 | 2.134 |
| Symmetry imposed |  | C14 | C16 | C1 | C1 | C1 |  | C5 | C1 | C1 | C1 |
| Initial particle images (no.) |  | 2,200,737 | | | | |  | 2,069,918 | | | |
| Final particle images (no.) |  | 784,923 | 784,923 | 296,445 | 533,530 | 28,902 |  | 104,720 | 115,034 | 234,578 | 65,173 |
| Map resolution (Å) |  | 2.46 | 2.69 | 3.18 | 2.93 | 3.05 |  | 2.97 | 6.22 | 3.83 | 4.33 |
| FSC threshold |  | 0.143 | 0.143 | 0.143 | 0.143 | 0.143 |  | 0.143 | 0.143 | 0.143 | 0.143 |
| Map resolution range (Å) |  | 2.2-2.8 | 2.5-2.8 | 2.7-3.6 | 2.5-3.4 | 2.6-4.5 |  | 2.3-3.3 | 4.0-8.0 | 3.2-5.0 | 3.5-7.0 |
| Map sharpening B factor (Å^2^) |  | -138 | -155.5 | -122.6 | -130.5 | -127.5 |  | -109.5 | -381.2 | -137.3 | -113.7 |
| Map was used to |  | Build the O-layer | Build the I-layer | Build the entire OMCC (Conformation-A) | Build the entire OMCC (Conformation-B) | Build the entire OMCC (Conformation-C) |  | Build the Stalk | Build the Arches asymmetric unit | Build the extended IMC protomer | Build the STALK-ARCHES-IMC |
| Part(s) built in the map |  | 14xTrwH/VirB7 14xTrwF/VirB9CTD 14xTrwE/VirB10O-layer | 16xTrwF/VirB9NTD 16xTrwE/VirB10I-layer | 14xTrwH/VirB7 14xTrwF/VirB9CTD 14xTrwE/VirB10O-layer 16xTrwF/VirB9NTD 16xTrwE/VirB10I-layer | 14xTrwH/VirB7 14xTrwF/VirB9CTD 14xTrwE/VirB10O-layer 16xTrwF/VirB9NTD 16xTrwE/VirB10I-layer | 14xTrwH/VirB7 14xTrwF/VirB9CTD 14xTrwE/VirB10O-layer 16xTrwF/VirB9NTD 16xTrwE/VirB10I-layer |  | 5xTrwJ/VirB5 5xTrwI/VirB6 | 4xTrwG/VirB8peri 2xTrwE/VirB10Arches | 1xTrwM/VirB3 2xTrwK/VirB4 1xTrwI/VirB6TM (A30-L82) 4xTrwG/VirB8tails 1xTrwE/VirB10IM-Cyto (Q21-V69) | 3xTrwM/VirB3 6xTrwK/VirB4 5xTrwJ/VirB5 5xTrwI/VirB6 8xTrwG/VirB8tails 3xTrwE/VirB10IM-CYTO 1xTrwE/VirB10ARCHES |
| Modelling method |  | Building and refinement | Building and refinement | Building and refinement | Building and refinement | Building and refinement |  | Building and refinement | AlphaFOLD and refinement | Building and refinement | Building and refinement |
| PDB deposited |  | 8RT4 | 8RT5 | 8RT6 | 8RT7 | 8RT8 |  | 8RT9 | 8RTA | 8RTB | 8RTD |

**B**. Model building and validation statistics.

| Complex/Subcomplex |  | **OMCC** | | | | |  | **STALK-ARCHES-IMC** | | | |
| --- | --- | --- | --- | --- | --- | --- | --- | --- | --- | --- | --- |
|  |  |  |  |  |  |  |  |  |  |  |  |
| Structures generated in this study |  | O-Layer | I-Layer | Conformation-A | Conformation-B | Conformation-C |  | Stalk | Arches asymmetric unit | Extended IMC protomer | STALK-ARCHES-IMC |
| PDB entry |  | 8RT4 | 8RT5 | 8RT6 | 8RT7 | 8RT8 |  | 8RT9 | 8RTA | 8RTB | 8RTD |
|  |  |  |  |  |  |  |  |  |  |  |  |
| **Refinement^1^** |  |  |  |  |  |  |  |  |  |  |  |
| EMDB corresponding |  | EMD-19478 | EMD-19479 | EMD-19480 | EMD-19481 | EMD-19482 |  | EMD-19483 | EMD-19484 | EMD-19485 | EMD-19488 |
| Initial model used (PDB code) |  | 7O3J | 7O3T | 8RT4 & 8RT5 | 8RT4 & 8RT5 | 8RT4 & 8RT5 |  | 7O3V | 7Q1V | 7OIU | 8RT9, 8RTA & 8RTB |
| Model resolution (Å) |  | 3 | 2.7 | 3.2 | 3 | 4 |  | 2.9 | 7.9 | 3.8 | 6.7 |
| FSC threshold |  | 0.5 | 0.5 | 0.5 | 0.5 | 0.5 |  | 0.5 | 0.5 | 0.5 | 0.5 |
| CC Model vs. Data (mask) |  | 0.82 | 0.84 | 0.81 | 0.79 | 0.85 |  | 0.80 | 0.58 | 0.77 | 0.54 |
|  |  |  |  |  |  |  |  |  |  |  |  |
| **Model composition** |  |  |  |  |  |  |  |  |  |  |  |
| Nonhydrogen atoms |  | 38,458 | 17,456 | 55,802 | 55,802 | 55,802 |  | 17,866 | 2,999 | 14,135 | 40,105 |
| Protein residues |  | 5,054 | 2,144 | 7,184 | 7,184 | 7,184 |  | 2,395 | 710 | 1,804 | 8,260 |
| Ligands |  | --- | --- | --- | --- | --- |  | --- | --- | --- | --- |
|  |  |  |  |  |  |  |  |  |  |  |  |
| **B factors (Å^2^)** |  |  |  |  |  |  |  |  |  |  |  |
| Protein |  | 47.7 (90.4 – 58.4) | 7.11 (75.21 – 24.70) | 0.43 (103 – 29.4) | 0.01 (117 – 37.9) | 55.8 (219 – 120) |  | 34.9 (98.7 – 49.9) | 0.57 (663 – 35.8) | 4.11 (70.7 – 27.4) | 0.57 (367 – 34.6) |
| Ligand |  | --- | --- | --- | --- | --- |  | --- | --- | --- | --- |
|  |  |  |  |  |  |  |  |  |  |  |  |
| **R.m.s. deviations** |  |  |  |  |  |  |  |  |  |  |  |
| Bond lengths (Å) |  | 0.003 | 0.007 | 0.01 | 0.009 | 0.006 |  | 0.007 | 0.011 | 0.006 | 0.014 |
| Bond angles (°) |  | 0.513 | 0.772 | 0.758 | 0.696 | 0.769 |  | 0.778 | 2.03 | 0.78 | 1.752 |
|  |  |  |  |  |  |  |  |  |  |  |  |
| **Validation^1^** |  |  |  |  |  |  |  |  |  |  |  |
| MolProbity score |  | 1.41 | 1.51 | 1.69 | 1.57 | 1.98 |  | 1.61 | 1.02 | 2.02 | 1.25 |
| Clashscore |  | 6.40 | 9.83 | 9.71 | 8.90 | 16.95 |  | 9.87 | 0.51 | 11.82 | 1.9 |
| Rotamer outliers (%) |  | 1.19 | 0 | 0.07 | 0.03 | 0.07 |  | 0 | 0 | 0 | 0 |
|  |  |  |  |  |  |  |  |  |  |  |  |
| **Ramachandran plot** |  |  |  |  |  |  |  |  |  |  |  |
| Favored (%) |  | 98.87 | 98.46 | 96.96 | 97.53 | 96.25 |  | 97.56 | 94.99 | 93.4 | 95.78 |
| Allowed (%) |  | 1.13 | 1.54 | 3.03 | 2.47 | 3.75 |  | 2.44 | 4.73 | 6.6 | 4.08 |
| Disallowed (%) |  | 0 | 0 | 0.01 | 0 | 0 |  | 0 | 0.29 | 0 | 0.14 |
