# Supplementary material for: Cryo-EM structure of a conjugative type IV secretion system suggests a molecular switch regulating pilus biogenesis
Source: EMBO J. 2024 Jun 17;43(15):10. doi: 10.1038/s44318-024-00135-z (PMC11294453; doi:10.1038/s44318-024-00135-z)
Supplement: Supplementary file 5 — Expanded View Figures [file 44318_2024_135_MOESM5_ESM.pdf]

Expanded View Figures

Figure EV1. Summary of maps and structure and workflow for OMCC maps.

(**A**) Summary of maps and structures. Top: unsharpened cryo-EM maps displayed. Sigma level at which the maps have been contoured are reported. Bottom: structures derived from maps and colour-coded as in Fig. 1. "Side chains" and "secondary structures" labels indicate which structures are reported with side and main chains, respectively. (**B**) Image processing workflow for the obtained OMCC maps. This panel provides details about the image processing workflow for the OMCC maps. It reports on all steps during processing Final sharpened maps coloured by local resolution are shown. FSC curve and angle distribution are reported.

© The Author(s)

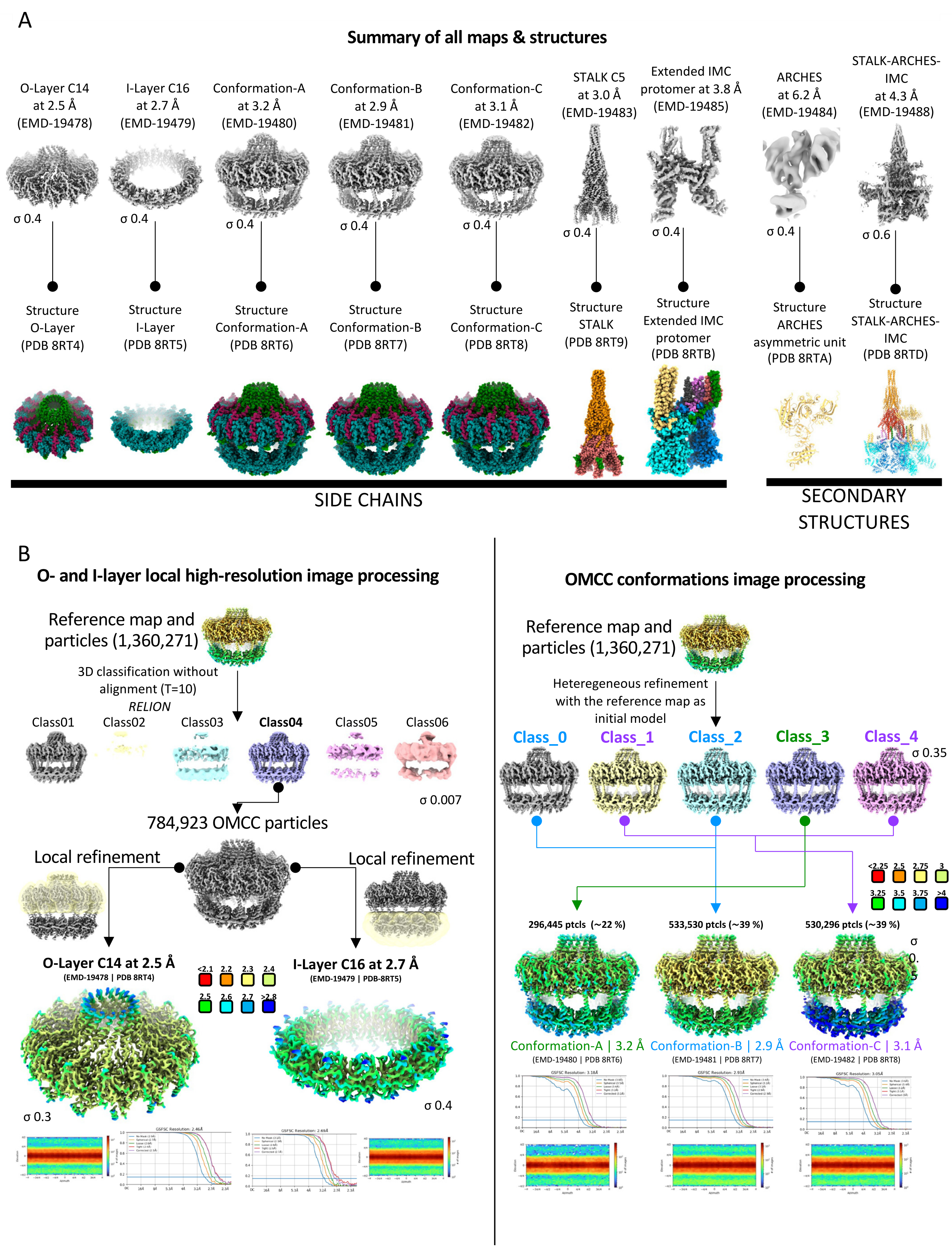
A
Summary of all maps & structures
O-Layer C14 at 2.5 Å (EMD-19478)
I-Layer C16 at 2.7 Å (EMD-19479)
Conformation-A at 3.2 Å (EMD-19480)
Conformation-B at 2.9 Å (EMD-19481)
Conformation-C at 3.1 Å (EMD-19482)
STALK C5 at 3.0 Å (EMD-19483)
Extended IMC protomer at 3.8 Å (EMD-19485)
ARCHES at 6.2 Å (EMD-19484)
STALK-ARCHES-IMC at 4.3 Å (EMD-19488)
σ 0.4
σ 0.6
Structure O-Layer (PDB 8RT4)
Structure I-Layer (PDB 8RT5)
Structure Conformation-A (PDB 8RT6)
Structure Conformation-B (PDB 8RT7)
Structure Conformation-C (PDB 8RT8)
Structure STALK (PDB 8RT9)
Structure Extended IMC protomer (PDB 8RTB)
Structure ARCHES asymmetric unit (PDB 8RTA)
Structure STALK-ARCHES-IMC (PDB 8RTD)
SIDE CHAINS
SECONDARY STRUCTURES
B
O- and I-layer local high-resolution image processing
Reference map and particles (1,360,271)
3D classification without alignment (T=10)
RELION
Class01 Class02 Class03 Class04 Class05 Class06
σ 0.007
784,923 OMCC particles
Local refinement
Local refinement
O-Layer C14 at 2.5 Å
(EMD-19478 | PDB 8RT4)
I-Layer C16 at 2.7 Å
(EMD-19479 | PDB-8RT5)
<2.1 2.2 2.3 2.4 2.5 2.6 2.7 >2.8
σ 0.3
σ 0.4
OMCC conformations image processing
Reference map and particles (1,360,271)
Heteregeneous refinement with the reference map as initial model
Class_0 Class_1 Class_2 Class_3 Class_4
σ 0.35
<2.25 2.5 2.75 3 3.25 3.5 3.75 >4
296,445 ptcls (~22 %)
533,530 ptcls (~39 %)
530,296 ptcls (~39 %)
σ 0.5
Conformation-A | 3.2 Å
(EMD-19480 | PDB 8RT6)
Conformation-B | 2.9 Å
(EMD-19481 | PDB 8RT7)
Conformation-C | 3.1 Å
(EMD-19482 | PDB 8RT8)

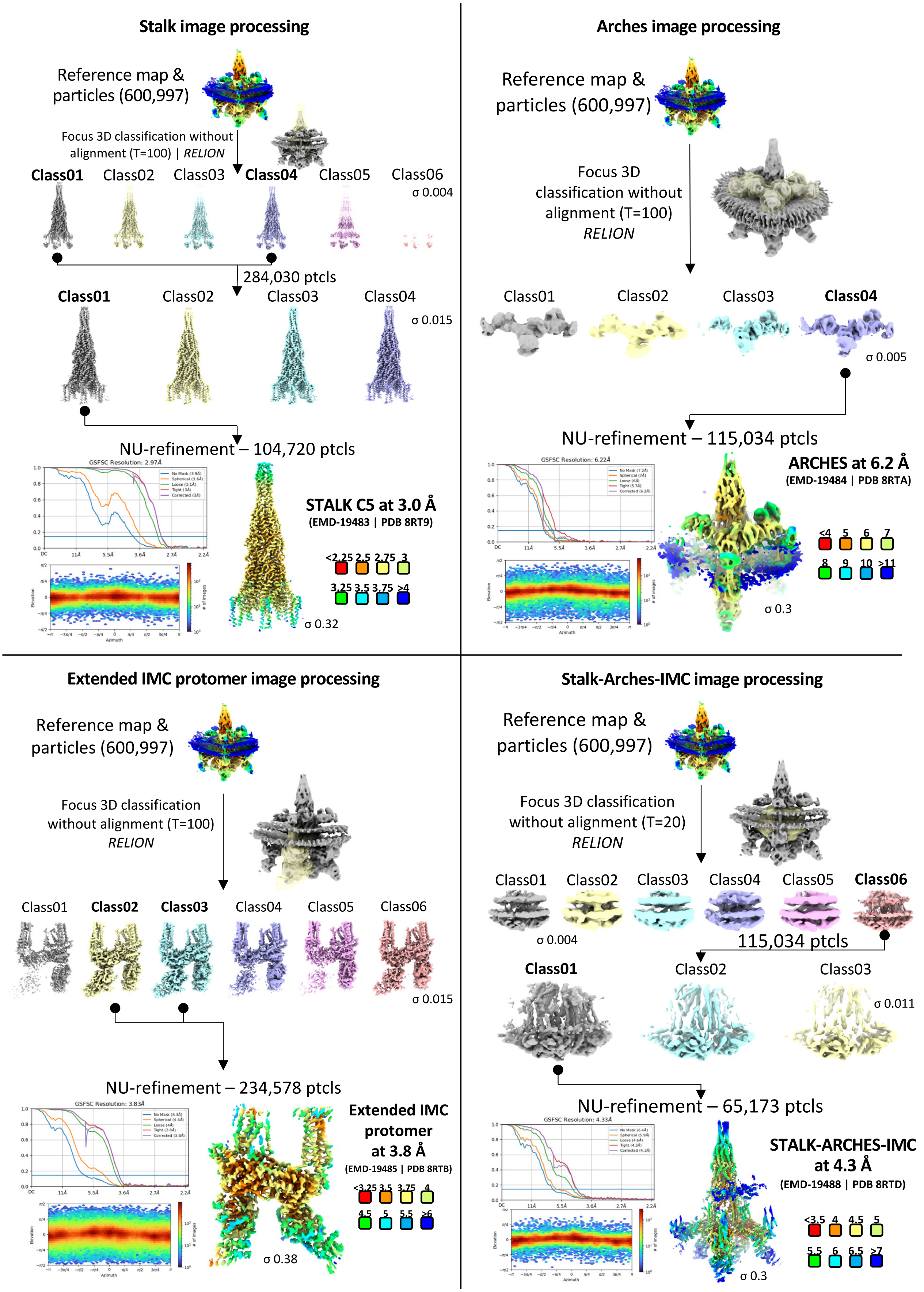

Stalk image processing
Reference map & particles (600,997)
Focus 3D classification without alignment (T=100) | RELION
Class01 Class02 Class03 Class04 Class05 Class06
σ 0.004
284,030 ptcls
Class01 Class02 Class03 Class04
σ 0.015
NU-refinement – 104,720 ptcls
STALK C5 at 3.0 Å
(EMD-19483 | PDB 8RT9)
<2.25 2.5 2.75 3
3.25 3.5 3.75 >4
σ 0.32
Arches image processing
Reference map & particles (600,997)
Focus 3D classification without alignment (T=100) RELION
Class01 Class02 Class03 Class04
σ 0.005
NU-refinement – 115,034 ptcls
ARCHES at 6.2 Å
(EMD-19484 | PDB 8RTA)
<4 5 6 7
8 9 10 >11
σ 0.3
Extended IMC protomer image processing
Reference map & particles (600,997)
Focus 3D classification without alignment (T=100) RELION
Class01 Class02 Class03 Class04 Class05 Class06
σ 0.015
NU-refinement – 234,578 ptcls
Extended IMC protomer at 3.8 Å
(EMD-19485 | PDB 8RTB)
<3.25 3.5 3.75 4
4.5 5 5.5 >6
σ 0.38
Stalk-Arches-IMC image processing
Reference map & particles (600,997)
Focus 3D classification without alignment (T=20) RELION
Class01 Class02 Class03 Class04 Class05 Class06
σ 0.004
115,034 ptcls
Class01 Class02 Class03
σ 0.011
NU-refinement – 65,173 ptcls
STALK-ARCHES-IMC at 4.3 Å
(EMD-19488 | PDB 8RTD)
<3.5 4 4.5 5
5.5 6 6.5 >7
σ 0.3

 © The Author(s)

Figure EV2. Image processing workflow for stalk, arches asymmetric unit, extended IMC protomer and stalk-arches-IMC.

This figure provides a detailed overview of the image processing workflow for the stalk, arches asymmetric unit, extended IMC protomer and stalk-arches-IMC cryo-EM maps. It reports on all steps during processing. Final sharpened maps coloured by local resolution are shown. FSC curve and angle distribution are reported.

© The Author(s)

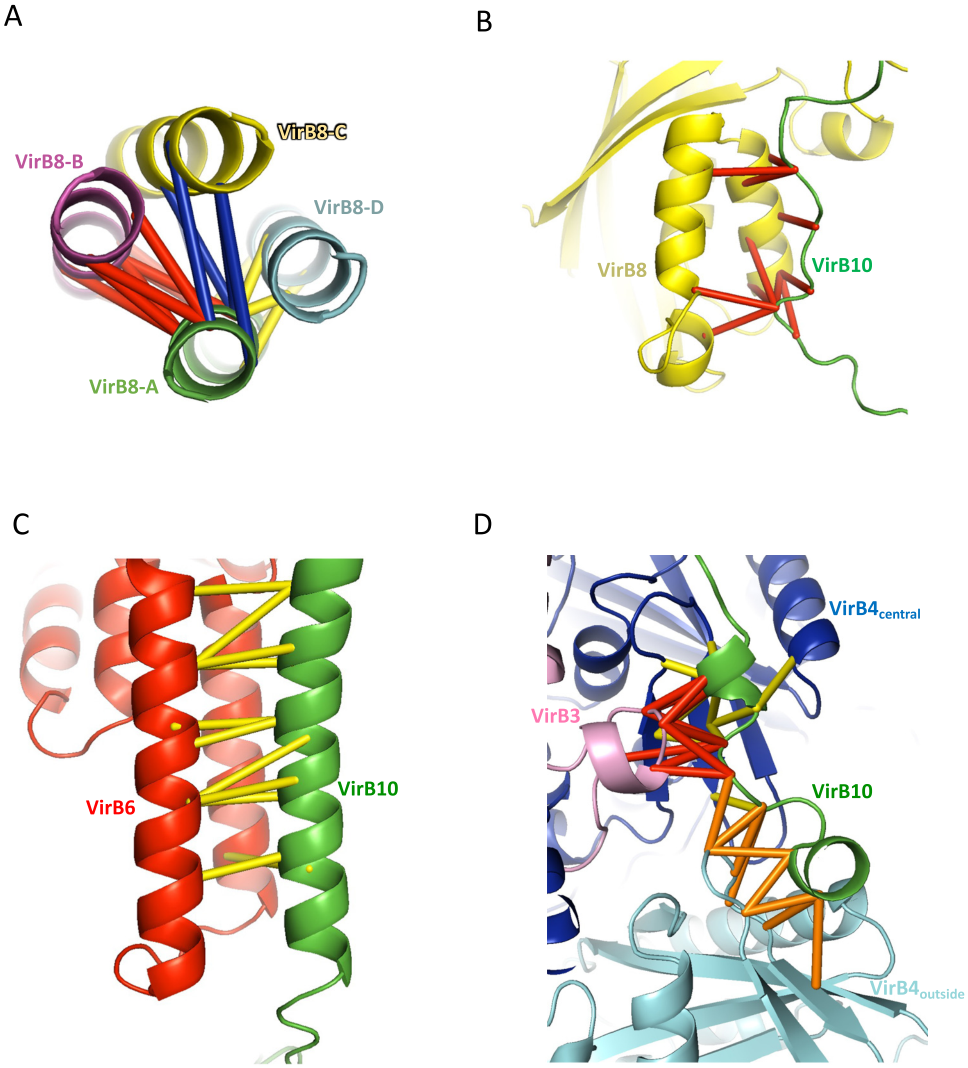

Figure EV3. AlphaFold models with top-scoring co-evolving residue pairs mapped.

(**A**) Co-evolving residues at the interface of VirB8-A with VirB8-B, VirB8-C and VirB8-D. The ten top-scoring residue pairs listed in Dataset EV1 are mapped for each interaction onto the corresponding Alphafold model. Colour coding for proteins is green, purple, yellow, and cyan blue for VirB8-A, VirB8-B, VirB8-C and VirB8-D, respectively. Residue pairs are shown by bars between their Calpha atoms, colour-coded red, blue, and yellow for VirB8-A and B, VirB8-A and C and VirB8-A and D interactions, respectively. (**B**) Co-evolving residues at the interface of VirB10 and VirB8.The ten top-scoring residue pairs in Dataset EV1 are mapped onto the corresponding Alphafold model. Colour coding for proteins is as in Fig. 1. Residue pairs are shown by red bars between their Calpha atoms. (**C**) Co-evolving residues at the interface of VirB10 and VirB6. The ten top-scoring residue pairs in Dataset EV1 are mapped onto the corresponding Alphafold model presented below. Colour coding for proteins is as in Fig. 1. Residue pairs are shown by yellow bars between their Calpha atoms. (**D**) Co-evolving residues at the interface of VirB10 with VirB3 and VirB4. The ten top-scoring residue pairs in Dataset EV1 are mapped for each interaction onto the corresponding Alphafold model. Colour coding for proteins is as in Fig. 1. Residue pairs are shown by bars between their Calpha atoms, colour-coded red, yellow and orange for VirB3-VirB10$_{NT}$, VirB4$_{central}$-VirB10$_{NT}$ and VirB4$_{outside}$-VirB10$_{NT}$ interactions, respectively.

 © The Author(s)
